# The enigma of the RING-UIM E3 ligases: its transformative impact on cancer research

**DOI:** 10.1186/s12967-025-07194-8

**Published:** 2025-10-14

**Authors:** Ye Wang, Yue Zhao, Qi Xin, Jihong Zhang

**Affiliations:** 1 Department of Pediatrics, Shengjing Hospital of China Medical University, Shenyang, China; 2 Hematology Laboratory, Shengjing Hospital of China Medical University, Shenyang, China

**Keywords:** RING-UIM E3 ligases, Cancer, Molecular mechanisms, Clinical significance

## Abstract

RING-UIM E3 ligases, a subfamily within the RING-type E3 ligases, comprise four members: RNF114, RNF125, RNF138, and RNF166. These ligases are crucial in various biological processes, including immunity, inflammation, epigenetics, and homologous recombination. Extensive research has demonstrated that RING-UIM E3 ligases fulfill specific biological roles in carcinogenesis by ubiquitinating critical oncogenes and tumor suppressors, thereby modulating various signaling pathways, differing their functions across distinct cancer contexts. This review comprehensively examines the multifaceted roles of RING-UIM E3 ligases in human cancers, elucidates the molecular mechanisms underpinning their actions and regulatory effects on cancer cells, and explores their potential clinical applications.

## Introduction

**Fig. 1:**
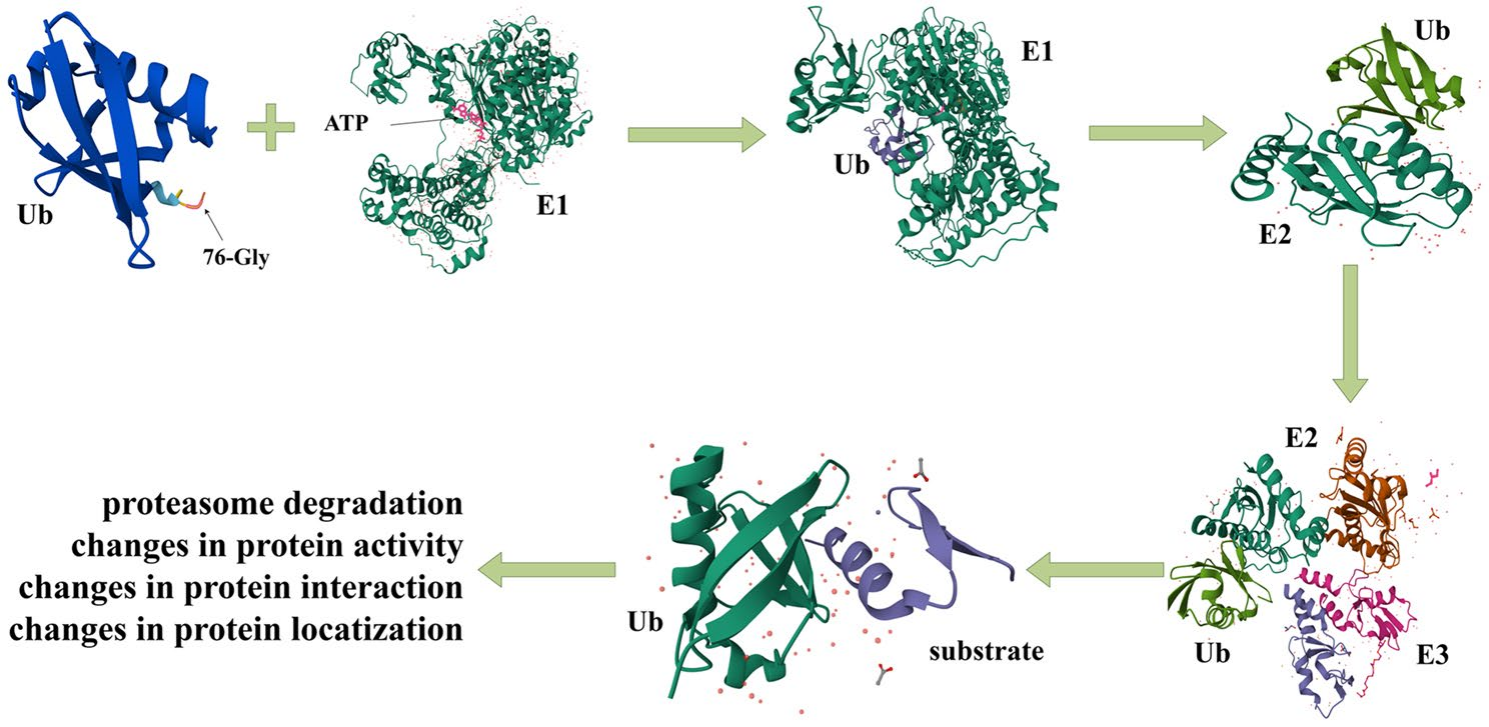
The process of ubiquitination. In the presence of ATP, ubiquitin conjugates with the E1 through the formation of a thioester bond, resulting in its activation. Following this, ubiquitin is transferred from the E1 to the E2 via a transesterification reaction. Ultimately, facilitated by the E3, the ubiquitin moiety on the E2-ubiquitin complex is transferred to the substrate protein, thereby modulating the substrate protein’s biological activity

Ubiquitination is a prevalent post-translational modification that regulates the activity of functional proteins in both spatial and temporal dimensions, thereby playing a pivotal role in the regulation of a diverse array of intracellular processes [1]. The ubiquitination process is facilitated through the sequential actions of three key enzymes: ubiquitin-activating enzyme (E1), ubiquitin-conjugating enzyme (E2), and ubiquitin ligase (E3). In summary, ubiquitin is initially activated through formatting a high-energy thioester bond with the E1 enzyme, a process that requires ATP as an energy source. Subsequently, the activated ubiquitin is transferred to the E2 enzyme, leading to the formation of the E2 ~ Ub conjugation. Finally, the E3 enzyme facilitates the transfer of ubiquitin from E2 to the target protein (Fig. 1) [2]. In ubiquitin molecules, the N-terminal methionine (Met-1) and seven internal lysine residues (K6, K11, K27, K29, K33, K48, and K63) function as acceptor sites for the sequential addition of ubiquitin, thereby facilitating the formation of a ubiquitin chain. Cells employ various ubiquitin linkage patterns as signaling mechanisms to determine the fate of proteins. For instance, ubiquitin chains that linked through K48 direct proteins towards proteasomal degradation, while that linked via K63 is associated with protein stabilization or involvement in diverse cellular signaling pathways [3].

In the process of ubiquitination, the E3 ligase serves as the pivotal enzyme within the ubiquitination reaction system [4]. To date, approximately 800 E3 ubiquitin ligases have been identified, and these E3 ligases are categorized into three principal groups— Homologous to E6AP C-terminus (HECT), Interesting new gene (RING), and RING-in-between-RING (RBR) —based on their distinctive structural domains and the mechanisms by which they transfer ubiquitin to substrate proteins [5, 6]. The RING-E3 class is the most prevalently and extensively researched among E3 ligases. It facilitates the adoption of a “closed” conformation by E2 enzymes, effectively positioning ubiquitin for nucleophilic attack on the substrate protein. Additionally, RING E3 functions as a scaffold, bringing E2 enzymes into proximity with their substrate proteins, thereby enhancing their interaction and promoting the subsequent transfer of ubiquitin from the E2 enzyme to the substrate [4].

**Fig. 2:**
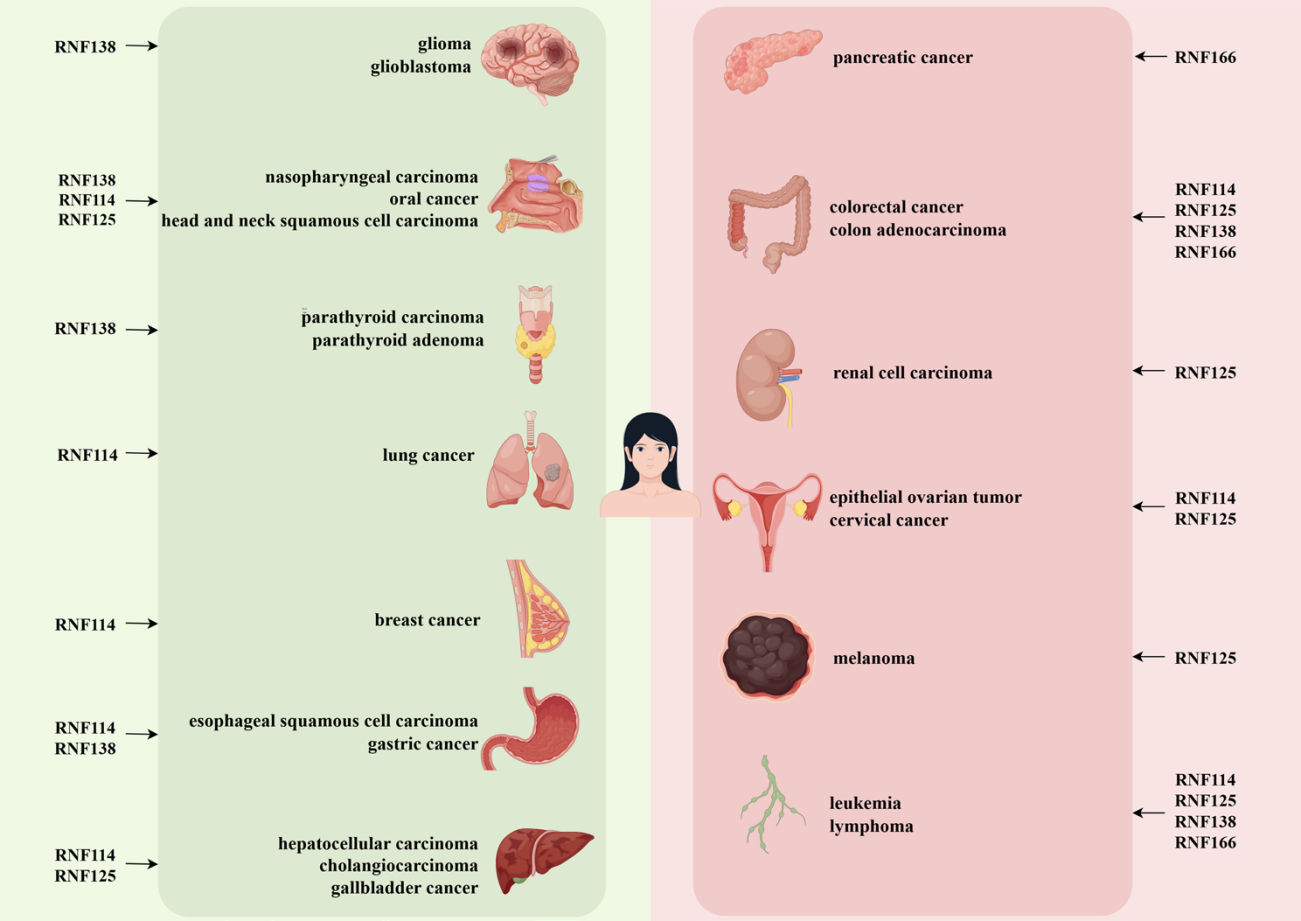
RING-UIM E3s in various cancers

RING-ubiquitin interaction motif (RING-UIM) E3 ligases represent a subset of RING-type E3 ligases, encompassing four distinct members: RNF114, RNF125, RNF138, and RNF166. RNF125 was initially discovered through functional screening aimed at identifying T cell regulatory factors. Subsequent investigations have demonstrated that RNF125 exhibits comparable protein size and structural characteristics to RNF114, RNF138, and RNF166, thereby establishing these proteins as a novel subfamily within the RING E3 ligases [7]. Despite comprising only four members, RING-UIM ligases are integral to various cellular processes, with their dysfunction related to neurodegenerative diseases, infections, inflammation, and cancer. In the context of cancer, the role of RING-UIM E3 ligases in the degradation of oncogenic or tumor-suppressive factors has been recognized as pivotal in the regulation of carcinogenesis of multiple human cancers (Fig. 2). This review seeks to thoroughly investigate the regulatory mechanisms of RING-UIM E3 ligases in oncogenesis, offering significant insights into recent advancements in this area and highlighting their potential significance in cancer therapy and prognostic evaluation.

## RING-UIM E3 ligase family

**Fig. 3:**
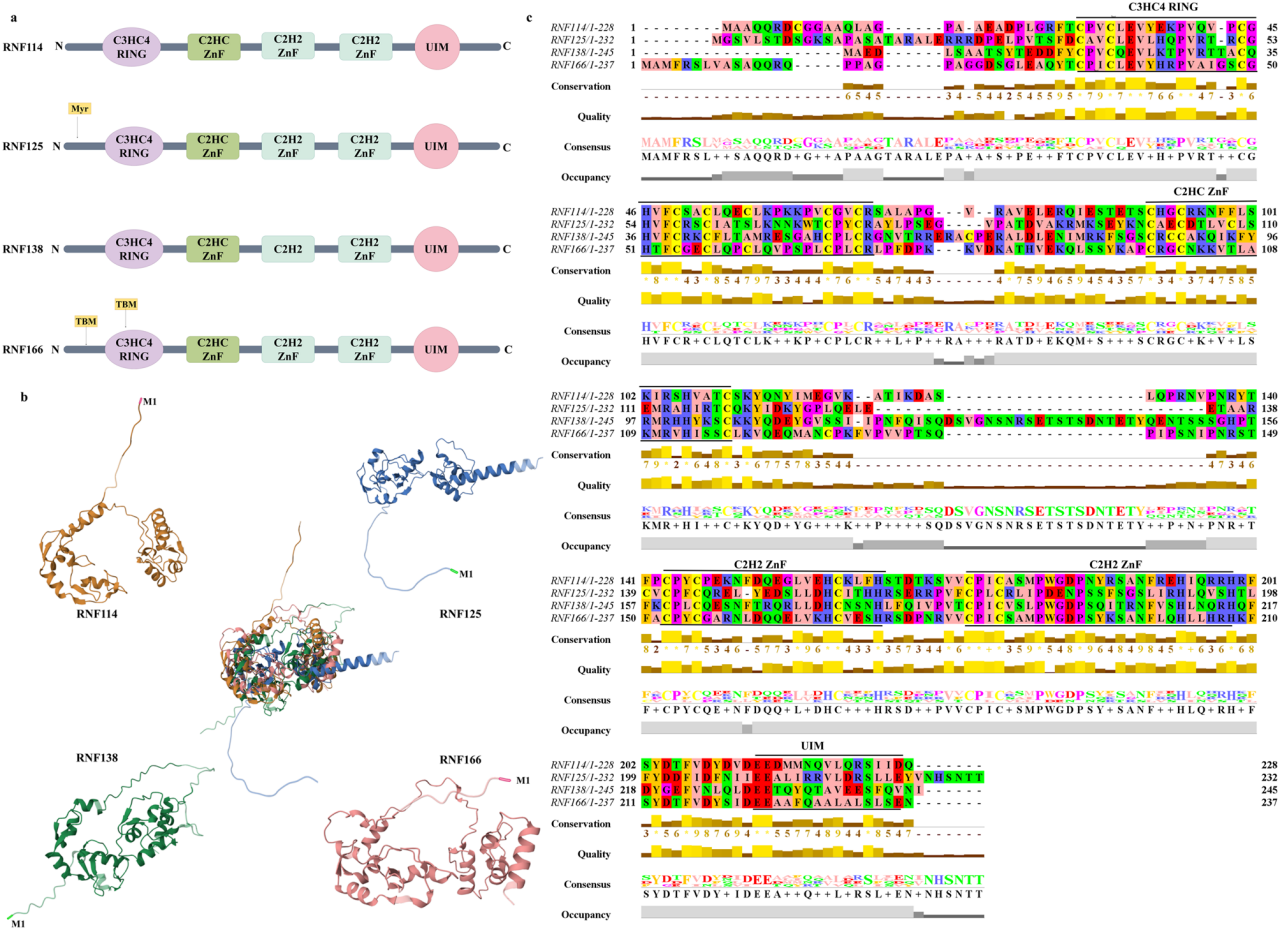
Structure diagram of the RING-UIM family. (**a**) Schematic diagram of the domains of RING-UIM family (RNF114, RNF125, RNF138, RNF166). The myristoylation site(Myr)of RNF125 and the TBMs of RNF166 is also marked with orange. (**b**) Three-dimensional structural superimposition diagram of the RING domains of RNF114, RNF125, RNF138 and RNF166. Methodology: Within the Pairwise Structure Alignment module located under the Analyze section of the RCSB PDB, input the respective UniProt IDs for RNF114 (Q9Y508), RNF125 (Q96EQ8), RNF138 (Q8WVD3), and RNF166 (Q96A37). Subsequently, select the Smith-Waterman 3D alignment method to acquire the three-dimensional structures and identify the overlapping patterns among these four proteins. (**c**) Multiple sequence alignment diagram of the RING–UIM family. Methodology: Enter the amino acid sequences of RNF114, RNF125, RNF138, and RNF166 into Clustal Omega to perform a sequence alignment of these four proteins. Following this, import the alignment results into Jalview to produce further sequence comparison analyses and visualize the amino acid sequences using the Zappo coloring scheme. Additionally, the amino acid sequences of the structural domains for each member of the RING-UIM family are labeled in the figure

The RING-UIM family represents a subfamily of RING-E3 ligases, comprising four members: RNF114, RNF125, RNF138, and RNF166. Members of this subfamily are characterized by five highly conserved structural domains: an amino-terminal C3HC4-RING domain, a central C2HC and two C2H2-type zinc fingers, and a carboxyl-terminal UIM [4, 7] (Fig. 3). The UIM domain as one of 15 distinct domains capable of binding ubiquitin is predominantly found in proteasome subunits, deubiquitinating enzymes, and proteins involved in receptor internalization [8, 9]. The RING-UIM E3 ligase plays a crucial role in the ubiquitination process: the C3HC4-RING domain recognizes the E2∼Ub conjugate, while the C-terminal UIM domain binds ubiquitin, facilitating its transfer to the substrate protein. During this process, the C2HC-ZnF domain stabilizes the RING domain and is implicated in substrate protein recognition [7, 10, 11].

RNF114, also referred to as ZNF313 or ZNF228, is a protein with a molecular weight of 25.7 kDa, characterized by an N-terminal RING structural domain, three zinc finger domains, and a C-terminal UIM [12, 13]. This soluble protein exhibits widespread expression across various tissues, with the highest levels observed in the testis, heart, liver, and kidney, and comparatively lower levels in skeletal muscle, lung, colon, brain, and placenta. Notably, RNF114 is localized in both the nucleus and cytoplasm of epithelial cells in normal tissues, whereas in cancer cells, it predominantly exhibits cytoplasmic distribution [14–17].

RNF125, also referred to as TRAC-1, is a small protein with a molecular weight of 25 kDa, exhibiting its highest expression levels in lymphoid tissues [7, 18–20]. In addition to possessing the common C3HC4-RING structural domain, zinc finger structural domain, and UIM structural domain, RNF125 is characterized by the presence of an N-terminal myristoylation site [10, 21]. The lipid modification at this myristoylation site is believed to facilitate the localization of RNF125 to the intracellular membrane. Furthermore, a short linker sequence (Li2 120–128) is known to enhance C2HC-Zn activity, while the C2HC-ZnF/Li2 120–128 region is crucial for the binding of the RING structural domain to the E2-binding enzyme [10].

RNF138, also referred to as HSD-4 or NARF, is a protein comprising 245 amino acids and characterized by the presence of an N-terminal C3HC4-RING structural domain, three zinc finger domains, and a C-terminal UIM domain [22, 23]. This protein is localized in both the cytoplasm and the nucleus and exhibits high expression levels in the testis and immune system [24–26]. RNF138 is implicated in various biological processes, including embryonic development, cell differentiation, proliferation, and regeneration. It plays a crucial role in maintaining chromosomal integrity and safeguarding genome stability, in addition to functioning as a negative regulator of inflammatory responses [27–31].

RNF166, a protein comprising 237 amino acids with an approximate molecular weight of 26,122 Da, is localized within both the cytoplasm and nucleus. It is composed of an N-terminal RING structural domain, three zinc finger domains, and a C-terminal UIM [32, 33]. Additionally, RNF166 contains two putative tankyrase-binding motifs (TBMs) [34]. This protein exhibits dual functionality, mediating the ubiquitination of Lys63 linkages and facilitating the SUMOylation of its cellular targets, suggesting potential E2/E3 enzymatic activity [32, 35]. Functionally, RNF166 is posited to play bifunctional roles in protein stability and degradation, depending on the cellular context and binding substrates [35, 36].

Proteins with physiological functions in the human body typically exhibit ordered structures, characterized by specific types and compositions of amino acids, precise amino acid sequences, and unique spatial arrangements of peptide chains. Figure 3c illustrates the differences in amino acid sequences among the four members of the RING-UIM family. Notably, all these proteins contain a C3HC4 RING structure near the N-terminus. While RNF138 and RNF166 display the conventional 40-residue spacing between cysteine and arginine pairs, RNF114 and RNF125 have a spacing of only 39 residues. Additionally, variations are observed in the number of amino acid residues within the first C2H2 ZnF domain of these proteins. Despite these differences, key residues within the shared domains remain conserved. Furthermore, the Pairwise Structure Alignment [37] of the RCSB PDB effectively delineates the similarities and differences in the three-dimensional structures of the four members of the RING-UIM family. Although similarities are present, the degree of overlapping regions differs (Fig. 3b). The unique amino acid sequences of these proteins are fundamental to their spatial conformation, resulting in variations in the three-dimensional structures among the four RING-UIM family members. The diverse amino acid sequences and specific spatial structures of the RING-UIM family enable the execution of distinct physiological functions that are vital for life.

## Regulatory mechanisms of RING-UIM E3 ligases in cancers

**Fig. 4:**
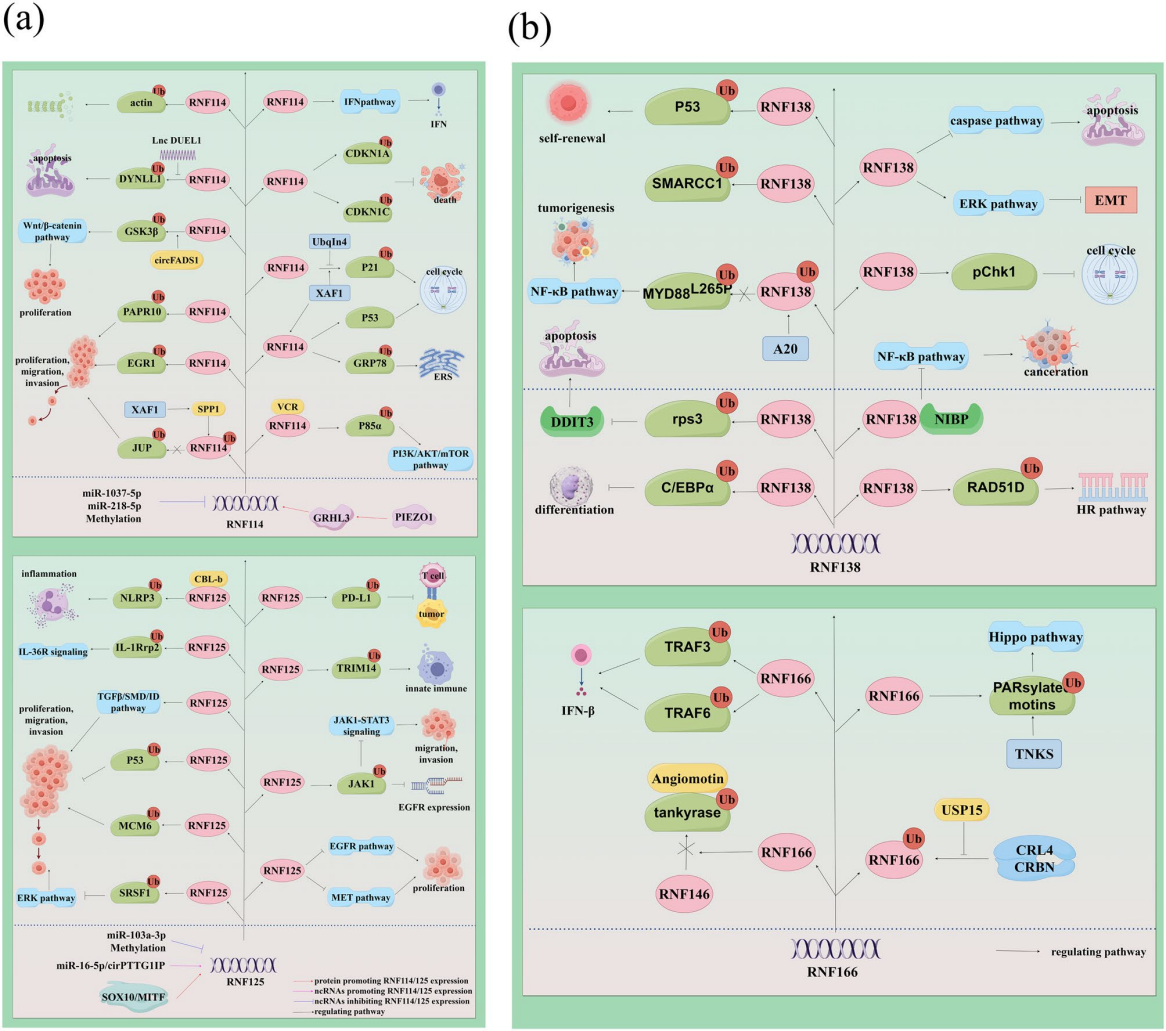
(**a**) The regulatory mechanisms governing RNF114 and RNF125 involve complex interactions with various molecular entities. The expression of RNF114 is modulated by miR-1037-5p, miR-8-5p, methylation processes, and the PIEZO1-GRHL3 axis. In contrast, RNF125 expression is influenced by the transcription factors SOX10/MITF, miR-16-5p/cirPTTG1IP, miR-103a-3p, and methylation. Both RNF114 and RNF125 contribute to the modulation of tumor cell behavior through the ubiquitination of substrate proteins and the regulation of multiple signaling pathways. Specifically, RNF114 facilitates the ubiquitination of a diverse array of substrate proteins, including JUP, EGR1, PAPR10, GSK3β, DYNLL1, actin, P85α, GRP78, CDKN1A, and CDKN1C, among others. The ubiquitination activity of RNF114 is either inhibited or enhanced by UbqIn4, Lnc DUEL1, circFADS1, XAF1, and SPP1, with XAF1 interacting with VCP to modulate RNF114’s ubiquitination function. Similarly, RNF125 catalyzes the ubiquitination of substrates such as SRSF1, MCM6, p53, NLRP3, JAK1, TRIM14, IL1Rrp2, and PD-L1, with CBL-b acting synergistically to enhance RNF125’s ubiquitination activity. (**b**) The regulatory roles of RNF138 and RNF166. RNF138 modulates the NF-κB signaling pathway through its interaction with NIBP, influences cell cycle progression by regulating pChk1, and impacts cellular biological behavior by facilitating the ubiquitination of RAD51D, C/EBPα, rpS3, MYD88^L256P^, SMARCC, and P53. Moreover, RNF138 is involved in the regulation of the ERK and caspase pathways. A20 also has the capacity to alter the ubiquitination status of RNF138. In contrast, RNF166 is implicated in the ubiquitination of PARsylated motins, TRAF3, and TRAF6, and it competitively inhibits the RNF146-mediated ubiquitination of tankyrase. Furthermore, RNF166 undergoes ubiquitination and deubiquitination processes mediated by CRL4/CRBN and USP15

Current research indicates that members of the RING-UIM family are pivotal in regulating cancer cell proliferation, apoptosis, migration, and invasion through their interaction with various substrates and signaling pathways (Fig. 4a-b; Table 1). Subsequent sections will elucidate the complex expression profiles, pathophysiological roles, and molecular functions of individual RING-UIM E3 ligases within cancerogenic contexts.

**Table 1:** Regulatory mechanisms, and clinical value of RING-UIM E3 ligases in cancer

Member	Cancer	Upstream	Substrate	Pathway / mechanism	Function	Clinical value	Reference
RNF114	colorectal cancer	XAF1/VCP	JUP	-	proliferation, migration, invasion	-	[70]
	gastric cancer	miR-218-5p、methylation	EGR1	-	proliferation, metastasis	-	[39]
		Ubqln4	P21	G1-S transition	proliferation	-	[69]
	cervical cancer	-	PARP10	-	migration, invasion	-	[40]
	breast cancer	-	CDKN1A, CDKN1C	-	-	treatment	[89]
	oral cancer	miR-1037-5p	-	-	-	prognosis, resistance	[66]
	esophageal squamous cell carcinoma	DLEU1	DYNLL1	Regulate BCL2	apoptosis	resistance	[67]
	hepatocellular carcinoma	circFADS1	GSK3β	Wnt/β-Catenin signaling	proliferation, apoptosis	prognosis, resistance	[68]
	non-small cell lung cancer	SPP1	P85α	PI3K/AKT/mTOR signaling	metastasis	-	[42]
RNF125	hepatocellular carcinoma	-		MET and EGFR signaling	proliferation	-	[43]
		miR-103a-3p	SRSF1	ERK signaling	proliferation, metastasis	-	[44]
		circPTTG1IP/miR-16-5p	JAK1	JAK1-STAT3 signaling	apoptosis, migration, invasion	treatment	[74]
		-	MCM6	-	proliferation	-	[45]
	head and neck squamous cell carcinoma	-	PD-L1	-	immune escape	-	[60]
	melanoma	SOX10/MITF	JAK1	EGFR signaling	-	resistance	[73]
	colorectal cancer	-	P53	-	proliferation	-	[46]
	gallbladder cancer	-	-	TGF-β1/SMAD3/ID1 signaling	migration, invasion	prognosis	[47]
RNF138	glioma	-	-		cell cycle, proliferation	-	[25, 48]
				Caspase signaling	apoptosis		
				ERK signaling	inhibit EMT		
	glioblastoma	-	-	P53/SOX2 signaling	self-renewal	-	[50]
		-	rpS3	DDIT3 signaling	apoptosis	radiotherapy	[94]
	leukemia	-	c/EBPα	-	myeloid differentiation	prognosis	[51]
	gastric cancer	-	-	Chk1 signaling	cell cycle	resistance	[92]
	colorectal cancer	-	NIBP	NF-κB signaling	canceration	-	[52]
	lymphoma	A20	MYD88^L265P^	NF-κB signaling	tumorigenesis	-	[49]
	nasopharyngeal carcinoma	-	-	attenuate DNA damage	-	resistance	[93]
RNF166	colorectal cancer		Motins	Hippo signaling	proliferation, migration	prognosis	[11]
	hematologic malignancy	USP15/CRL4-CRBN	-	-	-	resistance	[95]

### The ‘Double-Edged Sword’ in cancer proliferation, metastasis, and invasion

RNF114 is markedly overexpressed in colorectal cancer (CRC) and gastric cancer (GC). Its expression is significantly associated with the degree of cancer invasion, TNM stage, and overall survival in CRC patients. Silencing RNF114 expression has been shown to decrease proliferation and invasion of CRC cells while enhancing apoptosis, thereby effectively reducing cancer mass and volume, as well as lung metastasis, in an animal model [38]. RNF114 facilitates the degradation of downstream EGR1 via ubiquitination, thereby promoting GC cell proliferation, metastasis, and cancer growth in nude mice [39]. Furthermore, RNF114 positively modulates the functional activity of the cancer metastasis suppressor PARP10, which is involved in the migration and invasion of Hela cells [40, 41]. Additionally, SPP1 interacts with RNF114 to enhance the ubiquitination of P85α, which is implicated in regulating brain metastasis of non-small cell lung cancer via the PI3K/AKT/mTOR signaling pathway [42].

RNF125 is downregulated in hepatocellular carcinoma (HCC) and correlates with poor prognosis, exerting a negative regulatory effect on hepatocyte proliferation and liver regeneration by inhibiting the MET and EGFR pathways [43]. It also facilitates the proteasome-mediated degradation of SRSF1, thereby impeding HCC proliferation and metastasis through suppressing the ERK signaling pathway [44]. Furthermore, RNF125 interacts with MCM6, mediating its ubiquitination, which plays a crucial role in regulating HCC cell proliferation [45]. Additionally, the E3 ligase activity of RNF125 is essential for CRC cell growth, as its downregulation caused by the promoted ubiquitination and degradation of P53 may facilitate cancer growth [46]. While RNF125 inhibits cancerogenesis through various mechanisms, it also functions as a pro-cancerogenesis factor. In gallbladder cancer (GBC), RNF125 enhances the invasion and metastasis of GBC cells by activating the TGF-β1-SMAD3-ID1 signaling pathway [47].

The expression level of RNF138 in glioma tissues is markedly elevated compared to non-cancerous brain tissues. Down-regulation of RNF138 expression has been shown to inhibit proliferation and enhance apoptosis in glioma cell lines [25]. Another study reported that RNF138 knockdown increased apoptosis in glioma cells via the caspase pathway, reduced invasion and migration, and reversed EMT through ERK signaling [48]. Furthermore, aberrant localization of RNF138 was observed in glioma cells; in normal brain tissue, RNF138 colocalizes with endoplasmic reticulum markers but not nuclear markers, whereas in gliomas, it colocalizes with nuclear markers but not endoplasmic reticulum markers [24]. The relationship between this mislocalization and RNF138’s oncogenic role in gliomas remains unclear. In another study by Yu et al. [49] on lymphoma, RNF138 was found to promote lymphoma growth through catalyzing non-protective poly polyubiquitination of the MYD88^L265P^-K63 linkage, facilitating enhanced recruitment of IL-1 receptor-associated kinase and increased NF-κB activation, which is inhibited by A20.

The knockdown of RNF166 in CRC cell lines leads to marked reductions in cell proliferation, anchorage-dependent growth, and cell migration, as well as inhibiting cancer growth in vivo. Mechanistically, RNF166 contributes to the promotion of CRC cancer growth by recognizing and degrading PARsylated Motins, which leads to the activation of the Hippo pathway. Furthermore, the C-terminus of RNF166, particularly the Di19-ZF structural domain, is identified as a crucial region for the recognition of ADP-ribosylated angiopoietins. This domain represents a novel poly-ADP-nucleus-binding structure, which may serve as a potential therapeutic target [11].

### Maintenance of stem cell characteristics and disruption of cellular differentiation

Glioma stem cells (GSCs) are pivotal in the glioblastoma treatment, with RNF138 being highly expressed in these cells. The down-regulation of RNF138 has been shown to significantly decrease the expression of GSC markers CD133 and nestin. RNF138 may influence the self-renewal and cancerogenic potential of GSCs by modulating the p53 protein pathway and regulating its expression [50]. Furthermore, RNF114 can regulate the stemness of CRC cells and influence the malignant phenotype of CRC [38]. The expression of RNF138 is markedly elevated in bone marrow samples from patients with acute myelocytic leukemia (AML). This elevation contributes to the inhibition of myeloid differentiation in AML by ubiquitinating the cancer suppressor C/EBPα, which is associated with the overall survival rate of AML [51]. Additionally, research has demonstrated that RNF138-deficient mice exhibit a heightened susceptibility to the progression from colitis to aggressive malignant cancers. Further investigations have elucidated the pathogenic role of NF-κB signaling abnormalities resulting from the down-regulation of RNF138 in the progression from colitis to colon cancer transformation. These findings underscore the potential of targeting NF-κB signaling as a therapeutic strategy for specific CRC subtypes characterized by low RNF138 expression [52]. The RING-UIM E3 ligase may serve as a critical regulator of cancer cell stemness and differentiation. Targeting this ligase could offer therapeutic benefits by inhibiting cancer stem cells and addressing the issue of impaired cancer cell differentiation. Nonetheless, extensive basic and clinical research is required to further investigate this hypothesis.

### Immunoregulation

The overexpression of RNF114 has been shown to facilitate T cell activation and modulate signaling pathways that govern T cell-mediated immune responses [53, 54]. Research by Pang et al. [55] demonstrated that PIEZO1 upregulates the transcription factor GRHL3 in cytotoxic T cells derived from mouse cancer models and cancer patients. This upregulation induces the expression of RNF114, which interacts with filamentous actin, resulting in its downregulation and rearrangement, thereby inhibiting T cell traction. The administration of cytotoxic T cells treated with a PIEZO1 antagonist to cancer-bearing mice enhances T cell infiltration into the cancer tissues and suppresses cancer growth. Furthermore, RNF114 overexpression augments dsRNA-induced type I IFN production and modulates the type I IFN signaling pathway [14, 56].

RNF125 plays a novel role in the innate immune response by modulating the ubiquitination and subsequent degradation of TRIM14. Additionally, it influences the molecular interactions between IL-36R and factors involved in the inflammatory response and cell proliferation. The ubiquitination mediated by RNF125 and CBL-b is crucial for regulating the activation of NLRP3 inflammasomes [57–59]. According to Jiang et al. [60], overexpression of RNF125 facilitates the ubiquitin-mediated degradation of PD-L1 in head and neck squamous cell carcinoma (HNSCC) cells, thereby inhibiting immune evasion. Similarly, overexpression of RNF125 in MC-38 (mouse colon cancer) and H22 (mouse hepatocellular carcinoma) cell lines, when transplanted into mice, results in decreased PD-L1 levels, reduced cancer growth, and a significant increase in the infiltration of CD4+ T cells, CD8+ T cells, and macrophages [61].

Liu W et al. [31] demonstrated that RNF138 suppresses the transcription of late inflammatory genes by targeting SMARCC1 for degradation within the SWI/SNF complex. This study elucidates the intricate interactions among nucleosome remodeling, inflammation, and ubiquitination, highlighting the critical function of the E3 ubiquitin ligase in modulating the magnitude and persistence of the inflammatory response. Furthermore, RNF166 was shown to positively regulate IFN-β production through enhancing TRAF3 and TRAF6 ubiquitination [33].

In conclusion, RING-UIM E3 ligase is implicated in tumor immunity through regulating cytokine production, modulation of immune checkpoint expression, alteration of T-cell activity, and control of inflammation. However, there is scarce research concerning the interaction of RING-UIM E3 ligase with other immune checkpoints, such as CTLA-4 and LAG-3, as well as with immune cells, including macrophages and NK cells, within the tumor microenvironment. Consequently, further studies are warranted to elucidate the mechanisms by which RING-UIM E3 ligase influences tumor antigens and their gene expression, and to understand its role in the body’s immune surveillance and anti-tumor responses. Such research is essential for achieving the goal of inhibiting tumor immune evasion and advancing tumor treatment strategies.

### Others

RNF138 serves as a ubiquitin E3 ligase with a pivotal function in the regulation of the homologous recombination (HR) pathway. It is recruited to DNA damage sites via its zinc finger domain, where it facilitates DNA end resection and enhances ATR-dependent signaling, thereby promoting the repair of DNA double-strand breaks (DSBs) through HR [62]. Furthermore, RNF138 has been demonstrated to participate in the HR pathway by ubiquitinating RAD51D [23, 63]. Research has also identified RNF138 as a regulator of resection, with its activity being modulated by the complexity of DSBs across various cancer cell lines, which may potentially affect the fidelity of DSB repair in G1 phase cells [64].

RNF166 binds to and stabilizes ubiquitinated tankyrase in competition with RNF146-mediated degradation, and facilitates the bi-ubiquitination of K11 linkages. This process contributes to the stabilization of tankyrase and its interaction with its binding partner, the cancer cell signaling protein Angiomotin [34]. Additionally, RNF166 is a critical factor that interacts with autophagy-related proteins and plays a role in promoting apoptosis [36, 65].

## Regulation of RING-UIM E3 ligase

The expression and activity of RING-UIM E3 ligase family members are modulated by non-coding RNAs and various functional proteins (Fig. 4a-b; Table 1). The transcription factor GRHL3 is upregulated by PIEZO1, leading to the induction of RNF114 expression [55]. Furthermore, RNF114 expression is regulated by miR-1307-5p, miR-218-5p, and methylation modifications. Notably, miR-1307-5p facilitates the progression of oral cancer by inhibiting RNF114 [39, 66]. DLEU1 interacts with and stabilizes DYNLL1 by interfering with RNF114-mediated ubiquitin-proteasome degradation, thereby upregulating the anti-apoptotic protein BCL2 and promoting esophageal squamous cell carcinoma (ESCC) cells’ survival [67]. Additionally, circFADS1 associates with GSK3β and facilitates the ubiquitination and degradation of RNF114 by recruiting it to modulate the Wnt/β-Catenin signaling pathway [68]. Ubqln4 binds to RNF114 and stabilizes the function of p21 in cell cycle and cellular senescence regulation by negatively regulating RNF114 [69]. A recent study demonstrated that XAF1 enhances VCP-mediated ubiquitination of RNF114, which subsequently inhibits the ubiquitination-mediated degradation of JUP by RNF114, thereby influencing the migration and metastasis of CRC cells [70]. Furthermore, XAF1 facilitates the termination of p53-mediated cell cycle arrest through the activation of RNF114, intervenes in apoptosis, promotes cell cycle progression, and suppresses cellular senescence by stimulating the degradation of p21 WAF1 via RNF114 activation. Additionally, XAF1 induces an endoplasmic reticulum stress response through its interaction with RNF114 and the destabilization of the endoplasmic reticulum stress sensor GRP78 [15, 71, 72]. The SOX10/MITF axis enhances RNF125 expression as an upstream regulator [73]. Moreover, circPTTG1IP elevates RNF125 levels by binding to miR-16-5p, while RNF125 is also identified as a downstream target of miR-103a-3p [44, 74]. Furthermore, DNA methylation contributes to the reduced expression of RNF125 [75, 76].

## Clinical values of RING-UIM ligases

### Prognostic evaluation

Members of the RING-UIM E3 ligase family play a crucial role in regulating the biological characteristics of cancer cells and are associated with the prognosis of various cancers, making them valuable reference markers for clinical evaluation. Notably, RNF114 is overexpressed in GC and HCC with a 20q13.12-13.3 chromosomal gain, exhibiting a negative correlation with patient prognosis in GC and a significant association with vascular invasion in HCC and advanced cancer stages [39, 77]. RNF125, identified as a central gene in renal cell carcinoma (RCC) across different disease stages, is expressed at lower levels in RCC patients compared to healthy individuals, suggesting its potential as a biomarker for RCC [78]. Xiao et al. [79] utilized bioinformatics approaches to reveal that RNF125 is a key gene shared by pediatric acute lymphoblastic leukemia (ALL) and pediatric sepsis. It influences the immune environment differently in pediatric ALL and sepsis and holds significant diagnostic value for both conditions. Furthermore, gene-drug network analysis indicates that RNF125 may serve as a potential therapeutic target. RNF125 is significantly overexpressed in GBC tissues and is associated with Nevin staging and poor patient prognosis [47]. Conversely, RNF125 exhibits low expression in primary CRC and is further downregulated in colorectal liver metastases compared to healthy liver tissues. Notably, the relative expression level of RNF125 is elevated in CRC cell lines with high lymph node metastasis compared to those with low lymph node metastasis. Additionally, RNF125 expression is higher in patients with stage III CRC than in those with stage II [76, 80, 81]. According to Wang et al. [82], hsa_circ_0005729 does not correlate with RNF138 expression in parathyroid carcinoma patients, but shows a positive correlation in parathyroid adenoma patients. Furthermore, RNF138 expression is significantly upregulated in esophageal cancer tissues, yet markedly downregulated in CRC and colon adenocarcinoma (COAD) tissues. This downregulation is associated with COAD prognosis and serves as a prognostic marker with predictive value for hepatic metastasis in CRC [83–86]. The expression of RNF166 is elevated in CRC and pancreatic cancer, correlating with unfavorable patient prognosis. Additionally, RNF166 is linked to the infiltration of CD8 + T cells within pancreatic cancer tissue [11, 87].

### Potential for treatment

The natural anticancer compound Nimbolide has shown the ability to inhibit the E3 ligase activity of RNF114, resulting in the trapping of PARP1. This mechanism suggests the potential for targeting the RNF114-PARP1 pathway in the treatment of cancers deficient in homologous recombination. Furthermore, Nimbolide exhibits synthetic lethality with BRCA gene mutations, highlighting the promising prospect of utilizing Nimbolide and its analogs to target the RNF114-mediated PARP1 trapping pathway in BRCA-mutant cancers [88]. Additionally, Nimbolide can interfere with the recognition of endogenous RNF114 substrates, leading to the accumulation of such substrates, including the cancer suppressors CDKN1A and CDKN1C, thereby impairing breast cancer cell viability [89]. Moreover, Nimbolide contributes to the selective degradation of BCR-ABL in leukemia cells by recruiting RNF114 in proteolysis-targeting chimeras [90]. In ESCC cells, DLEU1 can bind and stabilize DYNLL1 to inhibit apoptosis by interfering with RNF114, whereas targeting DLEU1 enhances the sensitivity of ESCC cells to cisplatin treatment [67]. CircFADS interacts with GSK3β, facilitating its ubiquitination and subsequent degradation through recruiting the ubiquitin ligase RNF114. This interaction positions circFADS1 as a critical contributor to the promotion of lenvatinib resistance in HCC [68].

The expression of RNF125 is reduced in melanomas resistant to BRAF inhibitors, where it interacts with and ubiquitinates JAK1, leading to its degradation and the subsequent inhibition of EGFR expression. The concurrent inhibition of JAK and the EGFR markedly suppresses the proliferation of BRAF-resistant melanomas, suggesting its potential as a therapeutic strategy to counteract BRAF-resistant cancers [73]. Furthermore, RNF125 expression is elevated in chemotherapy-resistant epithelial ovarian cancers, although its precise regulatory mechanism remains unidentified [91]. Another study demonstrated that circPTTG1IP enhances RNF125 levels by binding to miR-16-5p, which in turn ubiquitinates and degrades JAK1 proteins. In contrast, Filgotinib, a JAK1 inhibitor, significantly impedes the progression of HCC, a result attributed to its inhibitory effect on the recruitment and polarization of cancer-associated macrophages [74].

The expression of RNF138 was found to be elevated in cisplatin-resistant GC cell lines, contributing to cisplatin resistance by mitigating cisplatin-induced, Chk1-mediated cell cycle arrest and apoptosis [92]. In nasopharyngeal carcinoma cells, RNF138 overexpression counteracted the effects of cisplatin on apoptosis and DNA damage, reduced the inhibitory impact of cisplatin on xenograft cancer growth, and led to significant enrichment of pathways associated with nasopharyngeal carcinoma progression and cisplatin resistance, such as the PI3K-Akt signaling pathway, human papillomavirus infection, and the ErbB signaling pathway [93]. Furthermore, in irradiated glioblastoma cells, rpS3 translocates to the nucleus, where RNF138 facilitates the ubiquitin-dependent degradation of rpS3. This process inhibits rpS3/DDIT3-mediated apoptotic signaling, contributing to radioresistance in glioblastoma cells [94].

Immunomodulatory drugs (IMiDs) have significantly advanced the treatment of multiple myeloma and other hematologic malignancies; however, resistance to IMiDs eventually develops in nearly all patients. Notably, USP15 is overexpressed in IMiD-resistant cells, and its depletion increases the sensitivity of these cells to lenalidomide. Furthermore, USP15 counteracts the ubiquitination of the CRL4-CRBN target protein RNF166, thereby inhibiting its degradation [95]. Nonetheless, it remains unclear whether USP15 modulates RNF166 through its deubiquitinating enzyme activity, influencing cancer cell resistance to IMiDs. This area warrants further investigation, as it presents a promising opportunity to enhance the therapeutic efficacy against hematologic malignancies.

## Discussion and perspective

In summary, RING-UIM E3 ligases are pivotal in the progression of human malignant cancers and significantly influence patient prognosis. Their roles are largely contingent upon cancer-specific substrates and associated signaling pathways. Moreover, the dysregulation of RING-UIM E3 ligases in cancers frequently involves the modulation of non-coding RNAs and specific transcription factors, necessitating the support of other functional proteins for their activity. The multifaceted and intricate functions of RING-UIM E3 ligases offer substantial potential for advancing the understanding of pathophysiological processes and cancers.

There have been a substantial body of research examining the roles of RNF114, RNF125, and RNF138 in neoplastic diseases. However, the role of RNF166 in cancer pathogenesis remains poorly understood. Notably, the expression and functional roles of RING-UIM E3 ligase members exhibit variability across different cancer types and even at various stages within the same cancer. Consequently, there is a pressing need for additional patient tissue samples to elucidate the expression patterns of RING-UIM E3 ligases and to investigate the molecular mechanisms influencing their expression and function. Such research could offer novel insights into their roles in cancer progression and potentially clinically significant discoveries. Furthermore, RING-UIM E3 ligases are not only pivotal in cancer immunology but also serve as mediators in the mechanisms underlying drug resistance in cancer cells. Therefore, the development of targeted agents based on the regulatory mechanisms of RING-UIM E3 ligases in cancers represents a challenging yet promising frontier in the field of cancer drug discovery.

Despite numerous studies elucidating the biological functions of RING-UIM E3 ligases in carcinogenesis, significant gaps remain in our understanding of their comprehensive role. Further investigation into the pathophysiological functions of these ligases can be achieved through the development of transgenic or knockout animal models, alongside the analysis of pathological evidence from human cancer samples. Advanced molecular biology techniques are essential for a detailed examination of the molecular mechanisms by which RING-UIM E3 ligases influence carcinogenesis and drug resistance. Proteomic approaches will be employed to identify novel substrates of RING-UIM E3 ligases, which will subsequently be validated through various molecular biology methods. Additionally, an in-depth investigation into the substrate selection process of RING-UIM E3 ligases is warranted. Understanding the regulatory mechanisms governing RING-UIM E3 ligase activity remains a critical question. It is anticipated that this review will inspire further research into the molecular mechanisms of RING-UIM E3 ligases and facilitate the translation of these findings into clinical applications, ultimately benefiting cancer patients.

## Data Availability

Not applicable.

## Abbreviations

Ub: Ubiquitin
RING-UIM: RING-Ub interacting motif
RNF114: RING finger protein family 114
RNF125: RING finger protein family 125
RNF138: RING finger protein family 138
RNF166: RING finger protein family 166
E1: Ubiquitin-activating enzyme
E2: Ubiquitin-conjugating enzyme
E3: Ubiquitin ligase
HECT: Homologous to E6AP C-terminus
RING: Interesting new gene
RBR: RING-in-between-RING
CRC: Colorectal cancer
GC: Gastric cancer
EGR1: Early growth response 1;PARP10:poly (ADP-ribose) polymerase family member 10;SPP1:secreted phosphoprotein 1;HCC: hepatocellular carcinoma
SRSF1: Serine/argininesplicingfactor1;MCM6:minichromosome maintenance complex component
GBC: Gallbladder cancer
TGF-β1: Transforming growth factor-β1;SMAD3:SMAD family member 3;ID1:inhibitor of differentiation and DNA binding-1
MYD88: Myeloid differentiation primary response protein 88;GSCs, Glioma stem cells
CD133: GSC markers cluster of differentiation 133;AML: acute myelocytic leukemia
C/EBPα: CCAAT/enhancer binding protein
PIEZO1: Piezo-type mechanosensitive ion channel component 1;GRHL3:grainyhead like transcription factor 3;IFN: interferon
TRIM14: Tripartite motif-containing 14;CBL-b: Casitas-B-lineage lymphoma protein-b
NLRP3: The nucleotide-binding oligomerization domain, leucine-rich repeat, and pyrin domain containing protein 3
PD-L1: Programmed death-ligand 1
HNSCC: Head and neck squamous cell carcinoma
MC-38: Mouse colon cancer cell lines
H22: Mouse hepatocellular carcinoma cell lines
NK: Natural killer cells
SWI/SNF: Switching or sucrose non-fermentable complex
TRAF3: TNF receptor-associated factor 3
TRAF6: TNF receptor-associated factor 6
CTLA-4: Cytotoxic T-lymphocyte-associated protein 4
LAG-3: Lymphocyte activation gene 3 protein
HR: Homologous recombination
DSBs: DNA double-strand breaks
RAD51D: RAD51 paralog D
DLEU1: lncRNA deleted in lymphocytic leukemia 1
DYNLL1: Dynein Light Chain LC8-Type 1
BCL2: Apoptosis Regulator
circFADS1: A novel circRNA
GSK3β: A key component of the Wnt/β-catenin pathway
ESCC: Esophageal squamous cell carcinoma
XAF1: X-linked inhibitor of apoptosis -associated factor 1
VCP: Valosin-containing protein
JUP: Junction plakoglobin
GRP78: Glucose-regulated protein 78
SOX10: SRY-Box transcription factor 10
MITF: Melanocyte inducing transcription factor
circPTTG1IP: A novel circRNA
RCC: Renal cell carcinoma
ALL: Acute lymphoblastic leukemia
COAD: Colon adenocarcinoma
PARP1: Poly (ADP-ribose) polymerase 1
CDKN1A: Cyclin Dependent Kinase Inhibitor 1 A
CDKN1C: Cyclin Dependent Kinase Inhibitor 1 C
JAK: Janus kinase
EGFR: Epidermal growth factor receptor
BRAF: Serine-threonine protein kinase
Chk1: Checkpoint kinase 1
rpS3: Ribosomal protein S3
DDIT3: DNA damage inducible transcript 3
IMiDs: Immunomodulatory drugs
USP15: Ubiquitin specific peptidase 15
CRL4-CRBN: CUL4-RBX1-DDB1-CRBN (CRL4CRBN) E3 ubiquitin ligase
